# High therapeutic efficacy of triplet therapy in unresectable or metastatic colorectal cancer and its optimal application strategies

**DOI:** 10.1007/s10147-026-03101-3

**Published:** 2026-06-25

**Authors:** Hiroki Sakuyama, Hiroyuki Okuyama, Yoriyuki Tahara, Sena Tsukamoto, Ikuhiro Kita, Kotone Nomura, Akitsu Murakami, Yoshihiro Okita, Hironaga Satake, Takeshi Kato, Akihito Tsuji

**Affiliations:** 1 Department of Medical Oncology, Kagawa University Hospital, Miki, Japan; 2 Department of Medical Oncology, Japanese Red Cross Society Himeji Hospital, Himeji, Japan; 3 Department of Medical Oncology, Kochi Medical School, Nankoku, Japan; 4 Department of Surgery, NHO Osaka National Hospital, Osaka, Japan

**Keywords:** Unresectable or metastatic colorectal cancer (mCRC), Chemotherapy, Triplet chemotherapy, FOLFOXIRI, Anti-VEGF antibody, Anti-EGFR antibody

## Abstract

Systemic chemotherapy for unresectable or metastatic colorectal cancer (mCRC) has improved clinical outcomes through substantial therapeutic advances. Triplet chemotherapy based on FOLFOXIRI (5-fluorouracil, leucovorin, oxaliplatin, and irinotecan) has achieved high response rates and deep tumor shrinkage. In combination with molecularly targeted agents, triplet therapy plus bevacizumab, an anti-vascular endothelial growth factor (VEGF) antibody, has demonstrated superior efficacy compared with doublet chemotherapy plus bevacizumab. In patients with *RAS/BRAF* wild-type tumors, combination therapy with anti-epidermal growth factor receptor (EGFR) monoclonal antibodies such as cetuximab and panitumumab has also shown improved survival outcomes, although toxicity remains a major concern. Although intensified treatment is associated with increased gastrointestinal and hematologic toxicity, improved supportive care and optimized dose-adjustment strategies have enhanced the feasibility of triplet therapy in routine clinical practice. This review outlines the historical development of triplet therapy in mCRC, summarizes the major clinical trials investigating triplet chemotherapy combined with molecularly targeted agents, and discusses direct comparisons between anti-VEGF- and anti-EGFR-based strategies. Particular emphasis is placed on practical considerations for clinical implementation, including toxicity management, dose optimization, and multidisciplinary supportive care.

## Introduction

Systemic chemotherapy for unresectable or metastatic colorectal cancer (mCRC) has undergone substantial evolution over the past several decades. This progress has been driven by the sequential development of cytotoxic agents, the integration of molecularly targeted therapies, and advances in supportive care that have expanded the feasibility of intensive treatment in routine clinical practice [1–4]. Doublet regimens such as FOLFOX (5-fluorouracil, leucovorin, and oxaliplatin) and FOLFIRI (5-fluorouracil, leucovorin, and irinotecan), in combination with molecularly targeted agents, including anti-vascular endothelial growth factor (VEGF) antibodies or anti-epidermal growth factor receptor (EGFR) antibodies for selected patients, are currently established as standard first-line treatments. These strategies have resulted in meaningful improvements in survival outcomes and form the backbone of contemporary first-line therapy for mCRC.

Among available regimens, triplet chemotherapy based on FOLFOXIRI (5-fluorouracil, leucovorin, oxaliplatin, and irinotecan) represents one of the most potent cytotoxic platforms. Compared with doublet regimens, triplet therapy achieves higher response rates and deeper tumor shrinkage, thereby offering particular benefit for patients with a high tumor burden or those requiring rapid disease control, including candidates for conversion therapy [5]. However, its widespread adoption has historically been limited by concerns regarding toxicity and treatment tolerability.

With advances in supportive care and optimized dose-modification strategies, triplet therapy has become increasingly feasible in routine clinical practice. In parallel, the addition of molecularly targeted agents has further expanded its therapeutic potential, while simultaneously increasing the complexity of treatment selection in real-world settings. This article reviews the historical development of triplet therapy in mCRC, summarizes key clinical trials combining triplet chemotherapy with molecularly targeted agents, and discusses head-to-head comparisons between anti-VEGF and anti-EGFR strategies. Particular emphasis is placed on practical considerations for clinical implementation, including toxicity management, dose optimization, and multidisciplinary supportive care.

## Historical development of triplet therapy in metastatic colorectal cancer

### Evolution from fluorouracil monotherapy to doublet regimens

The foundation of systemic chemotherapy for mCRC was established with fluorouracil (5-FU), which was introduced in the late 1950s [6]. Subsequent phase III trials demonstrated that the addition of irinotecan or oxaliplatin to 5-FU significantly improved survival compared with 5-FU alone, leading to the establishment of FOLFIRI and FOLFOX as standard regimens [7, 8]. The GERCOR study demonstrated that the sequence of FOLFIRI and FOLFOX did not significantly influence overall survival [9]. This finding suggested that cumulative exposure to all three cytotoxic agents over the disease course, rather than the specific order of administration, was a key determinant of long-term outcome. However, in real-world clinical practice, a substantial proportion of patients are unable to receive second-line therapy because of disease progression, declining performance status, or treatment-related toxicity.

### Rationale for upfront triplet exposure

To address the limitation of incomplete drug exposure, the concept of administering all three cytotoxic agents upfront led to the development of the FOLFOXIRI regimen. Early clinical studies demonstrated superior efficacy of FOLFOXIRI compared with doublet regimens, establishing the biological and clinical rationale for triplet therapy [5].

### Advances in supportive care enabling triplet therapy

Initial concerns regarding toxicity constrained the widespread adoption of triplet therapy. The ERBIRINOX study [10] and the studies conducted by Saridaki et al. [11] reported a notably high incidence of grade 3 or higher diarrhea, highlighting unresolved safety concerns. In later trials, modifications were introduced to improve tolerability, including irinotecan dose adjustment and omission of bolus 5-FU administration. One notable dosing strategy in the DEEPER trial was the clear definition of dose-reduction criteria for each agent according to the specific adverse events observed, which allowed safe treatment administration while maintaining dose intensity as much as possible [12]. Furthermore, weekly administration of Cmab facilitated earlier detection of adverse events and enabled timely treatment interruption or dose reduction. Over time, improvements in supportive care, including the optimization of antiemetic prophylaxis, proactive management of diarrhea and neutropenia, and standardized dose-modification strategies, have substantially improved tolerability and enabled its safe implementation in routine practice.

## Triplet therapy combined with molecularly targeted agents

### Triplet therapy plus anti-VEGF antibody (Bevacizumab)

The addition of bevacizumab, an anti-VEGF antibody, to cytotoxic chemotherapy represented a major milestone in the treatment of metastatic colorectal cancer [13, 14]. By targeting tumor angiogenesis, bevacizumab provided a broadly applicable strategy that could be combined with various chemotherapy backbones, including triplet regimens. The TRIBE trial demonstrated that FOLFOXIRI plus bevacizumab significantly improved response rates, progression-free survival, and overall survival compared with FOLFIRI plus bevacizumab [15]. These findings were subsequently confirmed in the TRIBE2 trial [16], which further supported the clinical benefit of upfront triplet therapy combined with bevacizumab. In Japan, the feasibility of FOLFOXIRI plus bevacizumab was evaluated in prospective studies such as the QUATTRO trial [16].

The JACCRO CC-11 study further refined dose optimization strategies tailored to Japanese patients, contributing important real-world evidence regarding safety and tolerability [17]. In addition, the development of CAPOXIRI (capecitabine, oxaliplatin, and irinotecan), which incorporates oral capecitabine in place of infusional fluorouracil, offered an alternative triplet platform [18]. This regimen aimed to improve convenience while maintaining efficacy, although careful management of gastrointestinal toxicity, particularly diarrhea, was required. Collectively, these studies position Triplet Therapy plus bevacizumab as a robust and broadly applicable triplet-based strategy with a favorable balance between efficacy and tolerability.

### Triplet therapy plus anti-EGFR antibodies

The combination of triplet chemotherapy with anti-EGFR antibodies has been explored as a strategy to maximize tumor response. Early single-arm studies, including POCHER, ERBIRINOX, and the Saridaki trial [10, 11, 20], demonstrated remarkably high response rates and increased rates of secondary resection. However, these impressive antitumor effects were accompanied by substantial toxicity, particularly gastrointestinal adverse events such as severe diarrhea. This toxicity profile likely reflects overlapping adverse effects associated with irinotecan-based chemotherapy and EGFR inhibition.

Subsequent phase II trials, including VOLFI and FOCULM [21, 22], further investigated triplet therapy combined with anti-EGFR antibodies in molecularly selected populations. These studies consistently showed enhanced response rates and deep tumor shrinkage in patients with *RAS*/*BRAF* wild-type tumors, albeit at the cost of increased treatment-related toxicity requiring careful clinical management. Taken together, triplet therapy combined with anti-EGFR antibodies represents a highly effective but toxicity-intensive approach. Its use should therefore be limited to carefully selected patients and managed in experienced centers with adequate supportive care infrastructure.

## Head-to-head comparisons: Anti-EGFR versus Anti-VEGF antibodies in triplet therapy

Direct comparisons between anti-EGFR and anti-VEGF antibodies in combination with triplet chemotherapy have been limited. Nevertheless, such comparisons are clinically important, as they address the optimal choice of partner drug when intensive cytotoxic backbones are selected. The DEEPER trial [12] provided a randomized phase II comparison of modified FOLFOXIRI combined with cetuximab versus bevacizumab in patients with *RAS* wild-type metastatic colorectal cancer. Although overall outcomes in the intention-to-treat population were broadly comparable, prespecified subgroup analyses demonstrated a clear advantage of cetuximab in patients with left-sided tumors, particularly with respect to depth of response and progression-free survival. These findings highlighted the relevance of tumor sidedness and molecular characteristics when selecting targeted agents in the context of triplet chemotherapy. Importantly, the DEEPER trial incorporated structured dose-modification criteria and supportive care protocols, demonstrating that triplet therapy combined with anti-EGFR antibodies can be delivered safely in experienced centers.

## Triplet versus doublet chemotherapy combined with Anti-EGFR antibodies

Whether intensification from doublet to triplet chemotherapy provides additional benefit when combined with anti-EGFR antibodies has been evaluated in several randomized trials. These studies have yielded nuanced results that underscore the complexity of treatment intensification in molecularly selected populations. The phase III TRIPLETE trial [23, 24] compared modified FOLFOXIRI plus panitumumab with FOLFOX plus panitumumab in patients with *RAS* wild-type metastatic colorectal cancer. This study failed to demonstrate superiority of the triplet regimen in terms of objective response rate or progression-free survival, although longer follow-up suggested a potential overall survival advantage. Similarly, the TRICE trial did not show a clear benefit of triplet therapy over doublet therapy in short-term efficacy endpoints [25]. Notably, treatment-related toxicity, particularly diarrhea and neutropenia, was more frequent with triplet regimens, emphasizing the need to carefully balance efficacy against tolerability. Collectively, these trials indicate that intensified chemotherapy does not uniformly translate into improved outcomes. Therefore, the use of triplet chemotherapy combined with anti-EGFR antibodies should be individualized, taking into account patient characteristics, treatment goals, and the ability to manage toxicity. A summary of selected trials discussed in this review is shown in Table 1, and the doses of each drug are shown in Table 2.

**Table 1:** Summary of efficacy data for triplet chemotherapy combinations

Study	Patient subset	Treatment	ORR, % (OR, 95% CI)	mPFS, months (HR, 95% CI)	mOS, months (2-y OS rate) (HR, 95% CI)	DpR, %	ETS, %
TRIBE trial [15]		FOLFOXIRI + BEV vs FOLFIRI + BEV	65 vs 54 (1.59, 1.10–2.28, *p* = 0.013)	12.3 vs 9.7 (0.77, 0.65–0.93, *p* = 0.006)	29.8 vs 25.8 (0.80, 0.65–0.98, *p* = 0.03)		
TRIBE2 [16]		FOLFOXIRI + BEV vs FOLFOX + BEV → FOLFIRI + BEV	62 vs 50 (1.61, 1.19–2.18, *p* = 0.0023)	(mPFS2) 19.2 vs 16.4 (0.87, 0.73–1.04, *p* = 0.11)	27.4 vs 22.5 (0.82, 0.68–0.98, *p* = 0.032)		
QUATTRO [17]		FOLFOXIRI + BEV	72.1 (95% CI 59.9–82.3)	14.1 (95% CI 11.5–17.3)	(76.5%) (95% CI 65.5–87.6%)		
JACCRO CC-11 [18]	*RAS*mt	mFOLFOXIRI + BEV	75.8 (95% CI 65.1–86.5)	11.5 (95% CI 9.5–14.0)	Not reported	49.2 (range; -28.7%-100%)	73.8
QUATTRO-II [19]		CAPOXIRI + BEV vs FOLFOXIRI + BEV	84.6 vs 76.5 (OR is not reported. *p* = 0.329)	10.9 vs 10.6 (1.114 0.695–1.784 *p* = 0.654)	(74.3% vs 65.5%) (0.771, 0.371–1.602, *p* = 0.485)	51.1 vs 46.6 (*p* = 0.252)	82.0 vs 71.4 (*p* = 0.241)
POCHER [20]	Liver meta	Cmab + chrono-IFLO	79.1 (66.9–91.2)	14 (11–17)	37 (21–53)		
ERBIRINOX [10]		Cmab + FOLFIRINOX	80.9	9.5 (7.6–10.4)	23.1 (22.6–not achieved)		
Saridaki studies [11]	*KRAS*wt	Cmab + mFOLFOXIRI	70 (53.6–86.4)	10.2 (7.1–13.4)	30.3 (18.8–41.9)		
VOLFI [21]	*RAS*wt	mFOLFOXIRI + Pmab vs mFOLFOXIRI	87.3 vs 60.6 (4.47, 1.61–12.38, *p* = 0.04)	9.7 vs 9.7 (1.07, 0.69–1.67, *p* = 0.76)	35.7 vs 29.8 (0.67, 0.41–1.11, *p* = 0.12)	58.9 vs 40.9 (*p* = 0.04)	85.7 vs 60.0 (*p* = 0.01)
FOCULM [22]	*BRAF*/*RAS*wt	mFOLFOXIRI + Cmab vs mFOLFOXIRI	95.5 vs 76.5 (difference 19.1; 95% CI, 17.4–36.4)	15.5 vs 14.2 (0.54, 0.30–0.95, *p* = 0.031)	Not reached vs 33.2 (*p* = 0.20)	56.1 vs 44.0 (*p* = 0.012)	
DEEPER [12]	*RAS*wt	mFOLFOXIRI + Cmab vs mFOLFOXIRI + BEV	71.1 vs 69.1 (*p* = 0.71)	13.8 vs 12.8 (0.89, *p* = 0.32)	28.4 vs 33.9 (0.94, *p* = 0.68)	57.3 vs 46.0 (*p* = 0.0029)	79.9 vs 74.7 (*p* = 0.27)
	*RAS*/*BRAF*wt + left-sided	mFOLFOXIRI + Cmab vs mFOLFOXIRI + BEV	83.6 vs 72.9 (*p* = 0.14)	15.3 vs 11.7 (0.68, 0.47–0.98, *p* = 0.036)	53.6 vs 40.2 (0.54, 0.32–0.91, *p* = 0.020)	63.6 vs 47.8 (*p* = 0.0003)	
TRIPLETE [23, 24]	*RAS*/*BRAF*wt	mFOLFOXIRI + Pmab vs FOLFOX + Pmab	73 vs 76 (0.87, 0.56–1.34, *p* = 0.526)	12.7 vs 12.5 (0.88, 0.70–1.11, *p* = 0.277)	41.4 vs 33.3 (0.79, 0.63–0.99, *p* = 0.049)	48 vs 47 (*p* = 0.845)	57 vs 58 (*p* = 0.878)
TRICE [25]	*RAS*/*BRAF*wt, liver meta	mFOLFOXIRI + Cmab vs FOLFOX + Cmab	84.7 vs 79.7 (0.70, 0.30–1.67, *p* = 0.42)	11.8 vs 13.4 (0.74, 0.50–1.11, *p* = 0.14)	Not reported	59.6 vs. 55.0 (*p* = 0.039)	80.6 vs 77.0 (*p* = 0.63)

ORR, objective response rate; OR, odds ratio; mPFS, modified progression-free survival; HR, hazard ratio; mOS, modified overall survival; OS, overall survival; DpR, depth of response; ETS, early tumor shrinkage; FOLFOXIRI, 5-FU, leucovorin, oxaliplatin, and irinotecan; BEV, bevacizumab; FOLFIRI, 5-FU, leucovorin and irinotecan; FOLFOX, 5-FU, leucovorin and oxaliplatin; mFOLFOXIRI, modified 5-FU, leucovorin, oxaliplatin and irinotecan; CAPOXIRI, capecitabine, oxaliplatin and irinotecan; meta, metastases; Cmab, cetuximab; Pmab: panitumumab

**Table 2:** Doses of triplet chemotherapy

Study	Treatment (triplet arm)	Triplet chemotherapy dose, mg/m^2^ (IV)		
		Irinotecan	Oxaliplatin	5-FU
TRIBE [15]	FOLFOXIRI + BEV	165	85	3200
TRIBE2 [16]	FOLFOXIRI + BEV	165	85	3200
QUATTRO [17]	FOLFOXIRI + BEV	165	85	3200
JACCRO CC-11 [18]	FOLFOXIRI + BEV	150	85	2400
POCHER [20]	Cmab + chrono-IFLO	130	80	2400
ERBIRINOX [10]	Cmab + FOLFIRINOX	180	85	2400 (+ 400 bolus)
Saridaki studies [11]	Cmab + mFOLFOXIRI	150	65	600 (day2 and 3) (+ 400 bolus)
VOLFI [21]	Pmab + mFOLFOXIRI	150	85	3000
FOCULM [22]	Cmab + mFOLFOXIRI	165	85	2800
DEEPER [12]	Cmab + mFOLFOXIRI	150	85	2400
TRIPLETE [23]	Pmab + mFOLFOXIRI	150	85	2400
TRICE [25]	Cmab + mFOLFOXIRI	150	85	2400

IV, intravenous injection; FOLFOXIRI, 5-FU, leucovorin, oxaliplatin, and irinotecan; BEV, bevacizumab; Cmab, cetuximab; FOLFIRINOX, 5-FU, leucovorin, oxaliplatin, and irinotecan; mFOLFOXIRI, modified 5-FU, leucovorin, oxaliplatin, and irinotecan; Pmab, panitumumab

## Primary tumor sidedness and treatment selection

Primary tumor sidedness is an important factor when considering the use of anti-EGFR antibodies. The FIRE-3 trial compared FOLFIRI plus Cmab with FOLFIRI plus BEV. In patients with *RAS*wt tumors, mOS was longer with Cmab in those with left-sided primary tumors (38.2 vs 28.2 months; HR 0.71, 95% CI 0.55–0.92; *p* = 0.01), whereas no benefit was observed in those with right-sided tumors (18.5 vs 23.0 months; HR 1.14, 95% CI 0.71–1.84; *p* = 0.60) [26, 27]. Similarly, the PARADIGM trial compared FOLFOX plus Pmab with FOLFOX plus BEV. In patients with left-sided primary tumors, mOS was 37.9 versus 34.3 months (HR 0.82, 95% CI 0.68–0.99; *p* = 0.03), favoring Pmab. In contrast, in patients with right-sided primary tumors, mOS was 20.2 versus 23.2 months (HR 1.09, 95% CI 0.79–1.51), and the benefit of anti-EGFR therapy was not clearly demonstrated [28]. These findings suggest that anti-EGFR antibodies are mainly beneficial in patients with left-sided primary tumors.

As described above, the DEEPER trial also showed the benefit of FOLFOXIRI plus Cmab in left-sided tumors. In addition, 88% of patients enrolled in the TRIPLETE trial had left-sided primary tumors, suggesting that FOLFOXIRI plus Pmab may also be more effective mainly in this population. Taken together with the efficacy of triplet plus BEV over doublet plus BEV, these findings suggest that triplet plus BEV is a reasonable option for patients with right-sided primary tumors, whereas triplet plus anti-EGFR antibodies may be more effective for those with left-sided primary tumors in *RAS/BRAF* wt mCRC. Furthermore, analyses from FIRE-3 and PARADIGM suggest that even some patients with right-sided tumors may benefit from anti-EGFR antibodies when broader molecular alterations beyond *RAS/BRAF* are considered. These findings indicate that treatment selection should not rely solely on primary tumor location, but should increasingly incorporate detailed molecular profiling. Advances in comprehensive genomic analysis may enable more precise personalized treatment beyond tumor sidedness [29, 30].

## Practical clinical implementation: supportive care and toxicity management

### Patient selection for triplet therapy

Appropriate patient selection is critical for the successful implementation of triplet chemotherapy. Candidates for triplet therapy should be carefully evaluated based on performance status, age, comorbidities, tumor burden, and the clinical need for rapid tumor shrinkage. In addition, molecular characteristics such as *RAS*/*BRAF* status and tumor sidedness should be incorporated into treatment decision-making. Triplet plus anti-EGFR antibody therapy is generally considered for patients with *RAS/BRAF* wt, left-sided tumors who are expected to tolerate intensive treatment. In particular, patients with an Eastern Cooperative Oncology Group performance status of 0–1 and preserved major organ function are regarded as appropriate candidates. With regard to age, treatment selection should not be based solely on chronological age, but should incorporate a comprehensive assessment, including geriatric evaluation when appropriate. In addition, given the favorable depth of response observed with triplet plus anti-EGFR antibody therapy, this approach may offer an advantage in achieving deeper tumor shrinkage and may be particularly beneficial in patients for whom marked tumor reduction is desired, although further investigation is warranted.

### Key toxicities and their management

Triplet chemotherapy is associated with a distinct toxicity profile, including gastrointestinal and hematologic adverse events. Diarrhea, neutropenia, and fatigue are the most common dose-limiting toxicities and require early recognition and proactive management. When combined with anti-EGFR antibodies, skin toxicity and electrolyte disturbances should also be anticipated and managed appropriately.

### Dose modification and treatment continuation criteria

Structured dose-modification strategies are essential to ensure the safe delivery of triplet therapy. The use of predefined dose-reduction and treatment-interruption criteria, such as those employed in the DEEPER trial [12], enables treatment intensity to be maintained while minimizing severe adverse events. Importantly, timely dose adjustment should be viewed as an integral component of treatment optimization rather than as a treatment failure. The safety profiles of the representative trials discussed in this review are summarized in Table 3.

**Table 3:** The safety data for selected anti–EGFR plus triplet chemotherapy combinations

Study	Treatment (anti–EGFR plus triplet arm)	Grade ≥ 3 neutropenia, %	Grade ≥ 3 febrile neutropenia, %	Grade ≥ 3 diarrhea, %	Grade ≥ 3 skin toxicities, %
POCHER [20]	Cmab + chrono-IFLO	7/6^a^	Not reported	94/36^a^	20/15^a^
ERBIRINOX [10]	Cmab + FOLFIRINOX	38.0	4.8	52.4	14.4
Saridaki studies [11]	Cmab + mFOLFOXIRI	23.3	3.3	53.3	3.3
VOLFI [21]	Pmab + mFOLFOXIRI	15.6	0	25.0	14.1
FOCULM [22]	Cmab + mFOLFOXIRI	31.3	3.0	7.5	0
DEEPER [12]	Cmab + mFOLFOXIRI	56.0	8.6	12.0	13.1
TRIPLETE [23]	Pmab + mFOLFOXIRI	32.0	5.0	23.0	19.0
TRICE [25]	Cmab + mFOLFOXIRI	44.1	4.4	5.9	0

FOLFOXIRI, 5-FU, leucovorin, oxaliplatin, and irinotecan; BEV, bevacizumab; Cmab, cetuximab; FOLFIRINOX, 5-FU, leucovorin, oxaliplatin, and irinotecan; mFOLFOXIRI, modified 5-FU, leucovorin, oxaliplatin, and irinotecan; Pmab, panitumumab

^a^ Before/after dose reduction;

### Importance of multidisciplinary supportive care

**Fig. 1:**
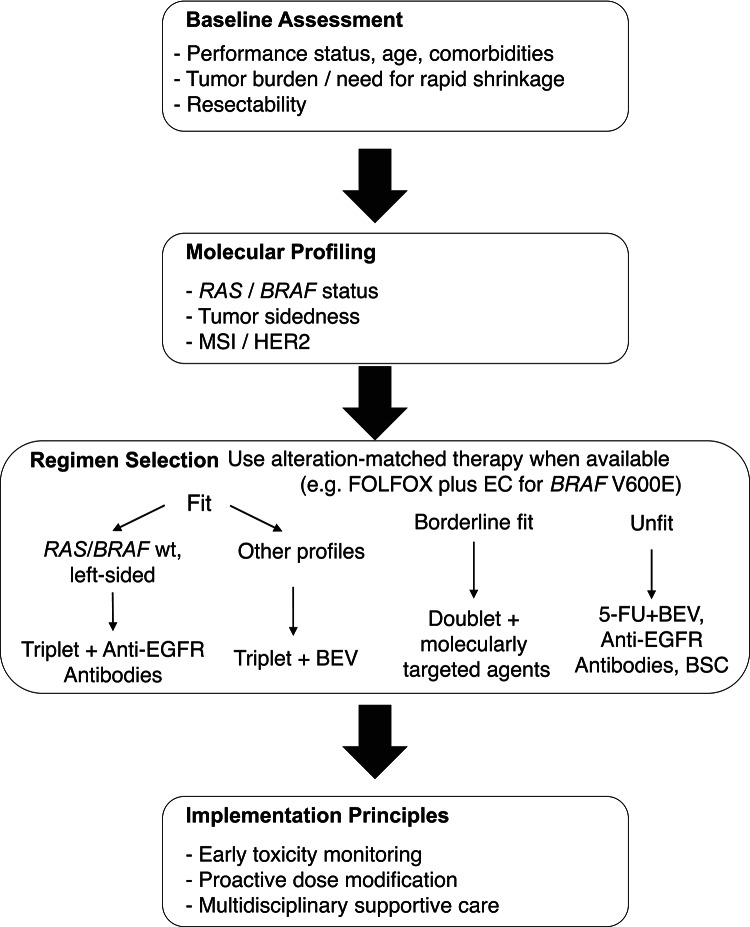
Clinical Algorithm for Triplet Therapy in Metastatic Colorectal Cancer. The proposed clinical algorithm for selecting and implementing triplet chemotherapy in metastatic colorectal cancer is shown. Treatment decisions should integrate patient fitness, tumor burden, molecular characteristics, and tumor sidedness, treatment goals, and the need for rapid tumor shrinkage. Accordingly, the chemotherapy backbone should be individualized, with careful consideration of treatment intensity and expected tolerability. Triplet chemotherapy with anti-EGFR antibody therapy should therefore not be regarded as a universal standard for all patients with left-sided *RAS/BRAF* wild-type disease. Alteration-matched therapy refers to treatment strategies selected according to specific genetic alterations, with the expectation of greater therapeutic efficacy, such as FOLFOX plus encorafenib and cetuximab for *BRAF* V600E alterations, or ipilimumab plus nivolumab for MSI-H tumors. In addition, the development of therapies targeting individual genetic alterations is ongoing, as exemplified by FOLFIRI plus sotorasib and panitumumab for *KRAS* G12C alterations in the CodeBreaK 301 trial. Therefore, it is necessary to continuously review and update the latest therapeutic options. Borderline fit refers to patients who may not tolerate highly intensive chemotherapy. This includes patients of advanced age with preserved functional status and those with mild concerns regarding comorbidities or organ function. Unfit refers to patients who are considered unlikely to tolerate chemotherapy. Examples include older patients, those with impaired organ function, and those with limited social or medical support. MSI, microsatellite instability; EC, encorafenib plus cetuximab; EGFR, epidermal growth factor receptor; BEV, bevacizumab; 5-FU, 5-fluorouracil; BSC, best supportive care

Effective supportive care is fundamental to the successful implementation of triplet chemotherapy. Close collaboration among oncologists, nurses, pharmacists, dietitians, and palliative care teams enables comprehensive toxicity management and supports treatment adherence. The treatment flow is shown in Fig. 1.

## Conclusion and future perspectives

Triplet chemotherapy remains one of the most potent cytotoxic platforms for the treatment of metastatic colorectal cancer. When appropriately combined with molecularly targeted agents and supported by meticulous toxicity management, it offers substantial clinical benefit for selected patients, particularly those requiring rapid and deep tumor shrinkage. At the same time, molecularly targeted therapy has advanced considerably in recent years. In patients with *BRAF* V600E-mutant disease, the efficacy of anti-EGFR antibodies had been considered limited based on the results of the FIRE-4.5 trial [31]. However, in light of the BRAKEWATER trial, FOLFOX plus encorafenib and cetuximab has now emerged as a promising treatment option [32]. In *RAS* mt, the efficacy of sotorasib plus panitumumab has been demonstrated in the CodeBreaK 300 trial, albeit currently only for *KRAS* G12C tumors, and its application in the first-line setting is now being explored [33]. Thus, ongoing development of therapies targeting specific molecular alterations is expected to further advance personalized medicine. In the future, combinations of these molecularly targeted agents with triplet chemotherapy may also be developed. As treatment strategies continue to evolve toward precision oncology, the integration of molecular profiling, tumor sidedness, and emerging multi-omics approaches is expected to further refine patient selection. Within this evolving landscape, triplet therapy is likely to retain a central role as a high-impact option, provided that its use is guided by careful patient selection and optimized supportive care.

## References

[1] Kinugasa Y, Uehara K, Yamaguchi K et al (2025) Japanese society for cancer of the colon and rectum (JSCCR) guidelines 2024 for the treatment of colorectal cancer. Int J Clin Oncol 30(12):2410–2463. 10.1007/s10147-025-02899-8 41186794 10.1007/s10147-025-02899-8 PMC12644182

[2] Morris VK, Kennedy EB, Baxter NN et al (2023) Treatment of metastatic colorectal cancer: ASCO guideline. J Clin Oncol 41(3):678–700. 10.1200/JCO.22.01690 36252154 10.1200/JCO.22.01690 PMC10506310

[3] Benson AB, Venook AP, Adam M et al (2024) Colon cancer, version 3.2024, NCCN clinical practice guidelines in oncology. J Natl Compr Canc Netw 22(2 D):e240029. 10.6004/jnccn.2024.0029 38862008 10.6004/jnccn.2024.0029

[4] Cervantes A, Adam R, Roselló S et al (2023) Metastatic colorectal cancer: ESMO clinical practice guideline for diagnosis, treatment and follow-up. Ann Oncol 34(1):10–32. 10.1016/j.annonc.2022.10.003 36307056 10.1016/j.annonc.2022.10.003

[5] Falcone A, Ricci S, Brunetti I et al (2007) Phase III trial of infusional fluorouracil, leucovorin, oxaliplatin, and irinotecan (FOLFOXIRI) compared with infusional fluorouracil, leucovorin, and irinotecan (FOLFIRI) as first-line treatment for metastatic colorectal cancer: the Gruppo Oncologico Nord Ovest. J Clin Oncol 25(13):1670–1676. 10.1200/JCO.2006.09.0928 17470860 10.1200/JCO.2006.09.0928

[6] Piedbois P, Rougier P, Buyse M et al (1998) Efficacy of intravenous continuous infusion of fluorouracil compared with bolus administration in advanced colorectal cancer. J Clin Oncol. 10.1200/JCO.1998.16.1.301 9440757 10.1200/JCO.1998.16.1.301

[7] Douillard JY, Cunningham D, Roth AD et al (2000) Irinotecan combined with fluorouracil compared with fluorouracil alone as first-line treatment for metastatic colorectal cancer: a multicentre randomised trial. Lancet 355(9209):1041–1047. 10.1016/s0140-6736(00)02034-1 10744089 10.1016/s0140-6736(00)02034-1

[8] de Gramont A, Figer A, Seymour M et al (2000) Leucovorin and fluorouracil with or without oxaliplatin as first-line treatment in advanced colorectal cancer. J Clin Oncol 18(16):2938–2947. 10.1200/JCO.2000.18.16.2938 10944126 10.1200/JCO.2000.18.16.2938

[9] Tournigand C, André T, Achille E et al (2004) FOLFIRI followed by FOLFOX6 or the reverse sequence in advanced colorectal cancer: a randomized GERCOR study. J Clin Oncol 22(2):229–237. 10.1200/JCO.2004.05.113 14657227 10.1200/JCO.2004.05.113

[10] Assenat E, Desseigne F, Thezenas S et al (2011) Cetuximab plus FOLFIRINOX (ERBIRINOX) as first-line treatment for unresectable metastatic colorectal cancer: a phase II trial. Oncologist 16(11):1557–1564. 10.1634/theoncologist.2011-0141 22016477 10.1634/theoncologist.2011-0141 PMC3233290

[11] Saridaki Z, Androulakis N, Vardakis N et al (2012) A triplet combination with irinotecan (CPT-11), oxaliplatin (LOHP), continuous infusion 5-fluorouracil and leucovorin (FOLFOXIRI) plus cetuximab as first-line treatment in *KRAS* wt, metastatic colorectal cancer: a pilot phase II trial. Br J Cancer 107(12):1932–1937. 10.1038/bjc.2012.509 23169296 10.1038/bjc.2012.509 PMC3516691

[12] Shiozawa M, Sunakawa Y, Watanabe T et al (2024) Modified FOLFOXIRI plus cetuximab versus bevacizumab in *RAS* wild-type metastatic colorectal cancer: a randomized phase II DEEPER trial. Nat Commun 15(1):10217. 10.1038/s41467-024-54460-2 39587053 10.1038/s41467-024-54460-2 PMC11589592

[13] Hurwitz H, Fehrenbacher L, Novotny W et al (2004) Bevacizumab plus irinotecan, fluorouracil, and leucovorin for metastatic colorectal cancer. N Engl J Med 3:2335–2342. 10.1056/NEJMoa032691 10.1056/NEJMoa032691 15175435

[14] Saltz LB, Clarke S, Díaz-Rubio E et al (2008) Bevacizumab in combination with oxaliplatin-based chemotherapy as first-line therapy in metastatic colorectal cancer: a randomized phase III study. J Clin Oncol 20:2013–2019. 10.1200/JCO.2007.14.9930 10.1200/JCO.2007.14.9930 18421054

[15] Cremolini C, Loupakis F, Antoniotti C et al (2015) FOLFOXIRI plus bevacizumab versus FOLFIRI plus bevacizumab as first-line treatment of patients with metastatic colorectal cancer: updated overall survival and molecular subgroup analyses of the open-label, phase 3 TRIBE study. Lancet Oncol 16(13):1306–1315. 10.1016/S1470-2045(15)00122-9 26338525 10.1016/S1470-2045(15)00122-9

[16] Cremolini C, Antoniotti C, Rossini D et al (2020) Upfront FOLFOXIRI plus bevacizumab and reintroduction after progression versus mFOLFOX6 plus bevacizumab followed by FOLFIRI plus bevacizumab in the treatment of patients with metastatic colorectal cancer (TRIBE2): a multicentre, open-label, phase 3, randomised, controlled trial. Lancet Oncol 21(4):497–507. 10.1016/S1470-2045(19)30862-9 32164906 10.1016/S1470-2045(19)30862-9

[17] Oki E, Kato T, Bando H et al (2018) A multicenter clinical phase II study of FOLFOXIRI plus bevacizumab as first-line therapy in patients with metastatic colorectal cancer: QUATTRO study. Clin Colorectal Cancer 17(2):147–155. 10.1016/j.clcc.2018.01.011 29530335 10.1016/j.clcc.2018.01.011

[18] Satake H, Sunakawa Y, Miyamoto Y et al (2018) A phase II trial of 1st-line modified-FOLFOXIRI plus bevacizumab treatment for metastatic colorectal cancer harboring *RAS* mutation: JACCRO CC-11. Oncotarget 10:18811–18820. 10.18632/oncotarget.24702 10.18632/oncotarget.24702 PMC5922357 29721163

[19] Bando H, Kotani D, Satake H et al (2024) QUATTRO-II randomized trial: CAPOXIRI+bevacizumab vs. FOLFOXIRI+bevacizumab as first-line treatment in patients with mCRC. Med 13:1164-1177.e3. 10.1016/j.medj.2024.05.012 10.1016/j.medj.2024.05.012 38901425

[20] Garufi C, Torsello A, Tumolo S et al (2010) Cetuximab plus chronomodulated irinotecan, 5-fluorouracil, leucovorin and oxaliplatin as neoadjuvant chemotherapy in colorectal liver metastases: POCHER trial. Br J Cancer 9:1542–1547. 10.1038/sj.bjc.6605940 10.1038/sj.bjc.6605940 PMC2990583 20959822

[21] Modest DP, Fischer von Weikersthal L, Decker T et al (2019) FOLFOXIRI plus panitumumab as first-line treatment of *RAS* wild-type metastatic colorectal cancer: the randomized, open-label, phase II VOLFI study (AIO KRK0109). J Clin Oncol 37(35):3401–3411. 10.1200/JCO.19.01340 31609637 10.1200/JCO.19.01340

[22] Hu H, Wang K, Huang M et al (2021) Modified FOLFOXIRI with or without cetuximab as conversion therapy in patients with *RAS*/*BRAF* wild-type unresectable liver metastases colorectal cancer: the FOCULM multicenter phase II trial. Oncologist 26(1):e90–e98. 10.1634/theoncologist.2020-0563 33400355 10.1634/theoncologist.2020-0563 PMC7794191

[23] Rossini D, Antoniotti C, Lonardi S et al (2022) Upfront modified fluorouracil, leucovorin, oxaliplatin, and irinotecan plus panitumumab versus fluorouracil, leucovorin, and oxaliplatin plus panitumumab for patients with *RAS/BRAF* wild-type metastatic colorectal cancer: the phase III TRIPLETE study by GONO. J Clin Oncol 1:2878–2888. 10.1200/JCO.22.00839 10.1200/JCO.22.00839 PMC9426812 35666229

[24] Conca V, Rossini D, Antoniotti C et al (2026) Upfront modified FOLFOXIRI plus panitumumab for *RAS/BRAF* wild-type metastatic colorectal cancer: final results of the phase III TRIPLETE study. J Clin Oncol 10:361–369. 10.1200/JCO-25-01337 10.1200/JCO-25-01337 PMC12879191 41505697

[25] Wang DS, Ren C, Li SS et al (2024) Cetuximab plus FOLFOXIRI versus cetuximab plus FOLFOX as conversion regimen in *RAS*/*BRAF* wild-type patients with initially unresectable colorectal liver metastases (TRICE trial): a randomized controlled trial. PLoS Med 10:e1004389. 10.1371/journal.pmed.1004389 10.1371/journal.pmed.1004389 PMC11086847 38728364

[26] Heinemann V, von Weikersthal LF, Decker T et al (2014) FOLFIRI plus cetuximab versus FOLFIRI plus bevacizumab as first-line treatment for patients with metastatic colorectal cancer (FIRE-3): a randomised, open-label, phase 3 trial. Lancet Oncol 15(10):1065–1075. 10.1016/S1470-2045(14)70330-4 25088940 10.1016/S1470-2045(14)70330-4

[27] Heinemann V, von Weikersthal LF, Decker T et al (2021) FOLFIRI plus cetuximab or bevacizumab for advanced colorectal cancer: final survival and per-protocol analysis of FIRE-3, a randomised clinical trial. Br J Cancer 124(3):587–594. 10.1038/s41416-020-01140-9 33154570 10.1038/s41416-020-01140-9 PMC7851157

[28] Watanabe J, Muro K, Shitara K et al (2023) Panitumumab vs bevacizumab added to standard first-line chemotherapy and overall survival among patients with *RAS* wild-type, left-sided metastatic colorectal cancer: a randomized clinical trial. JAMA 329(15):1271–1282. 10.1001/jama.2023.4428 37071094 10.1001/jama.2023.4428 PMC10114040

[29] Weiss L, Stintzing S, Stahler A et al (2025) Molecular hyperselection for optimal choice of first-line targeted therapy independent of primary tumor sidedness: an exploratory analysis of the randomized FIRE-3 study performed in RAS wild-type metastatic colorectal cancer. Eur J Cancer 15:115399. 10.1016/j.ejca.2025.115399 10.1016/j.ejca.2025.115399 40222201

[30] Shitara K, Muro K, Watanabe J et al (2024) Baseline ctDNA gene alterations as a biomarker of survival after panitumumab and chemotherapy in metastatic colorectal cancer. Nat Med 30(3):730–739. 10.1038/s41591-023-02791-w 38347302 10.1038/s41591-023-02791-w PMC10957476

[31] Stintzing S, Heinrich K, Tougeron D et al (2023) FOLFOXIRI plus cetuximab or bevacizumab as first-line treatment of *BRAF*^V600E^-mutant metastatic colorectal cancer: the randomized phase II FIRE-4.5 (AIO KRK0116) study. J Clin Oncol 1:4143–4153. 10.1200/JCO.22.01420 10.1200/JCO.22.01420 37352476

[32] Elez E, Yoshino T, Shen L et al (2025) Encorafenib, Cetuximab, and mFOLFOX6 in BRAF-Mutated Colorectal Cancer. N Engl J Med 392(24):2425–2437. 10.1056/NEJMoa2501912 40444708 10.1056/NEJMoa2501912 PMC12197837

[33] Fakih MG, Salvatore L, Esaki T et al (2023) Sotorasib plus Panitumumab in Refractory Colorectal Cancer with Mutated *KRAS* G12C. N Engl J Med 389(23):2125–2139. 10.1056/NEJMoa2308795 37870968 10.1056/NEJMoa2308795

